# The Role of Hydrogen Sulfide in the Pathophysiology and Therapeutic Modulation of Hypertension: A Scoping Review

**DOI:** 10.7759/cureus.109529

**Published:** 2026-05-23

**Authors:** Nikolay N Nikolov, Stefan N Yambolov, Rozen K Grigorov, Yanko G Yankov

**Affiliations:** 1 Faculty of Medicine, Medical University "Prof. Dr. Paraskev Stoyanov", Varna, BGR; 2 Department of Interventional Cardiology, University Hospital "St. Marina", Varna, BGR; 3 First Department of Internal Medicine, Medical University "Prof. Dr. Paraskev Stoyanov", Varna, BGR; 4 Clinic of Maxillofacial Surgery, University Hospital "St. Marina", Varna, BGR; 5 Department of General and Operative Surgery, Medical University "Prof. Dr. Paraskev Stoyanov", Varna, BGR

**Keywords:** aged garlic extract, blood pressure regulation, endothelial dysfunction, gasotransmitters, hydrogen sulfide, hypertension, nitric oxide signaling, oxidative stress, therapeutic modulation, vascular remodeling

## Abstract

Hydrogen sulfide (H₂S) is an endogenous gasotransmitter involved in vascular regulation, endothelial signaling, oxidative balance, and inflammatory pathways relevant to blood pressure control. Experimental and clinical evidence suggest that reduced H₂S bioavailability contributes to hypertension and associated vascular dysfunction. This study aimed to map and synthesize the breadth of mechanistic, preclinical, and clinical evidence on the role of H₂S in the pathophysiology and therapeutic modulation of hypertension. This scoping review followed the Preferred Reporting Items for Systematic Reviews and Meta-Analyses extension for Scoping Reviews (PRISMA-ScR) guideline. A comprehensive search of PubMed was conducted for English-language publications from January 1, 2001, to July 31, 2025, with a final update on August 10, 2025. Supplementary sources included Google Scholar (first 300 relevance-ranked results screened) and ClinicalTrials.gov. Prespecified Boolean strategies were used to identify mechanistic studies, animal experiments, human observational studies, interventional trials, and relevant secondary literature. Data were charted using a standardized extraction framework. A total of 873 records were identified. After removing 223 duplicates, 650 unique records underwent title/abstract screening; 69 full texts were assessed; and 39 sources were included. Mechanistic and preclinical data consistently demonstrated that reduced H₂S signaling leads to endothelial dysfunction, oxidative stress, vascular remodeling, and elevated blood pressure, while H₂S supplementation improves vascular tone. Human observational studies showed lower circulating H₂S levels and impaired H₂S-mediated vasodilation in hypertensive populations. Interventional trials using H₂S-enhancing strategies, particularly aged garlic extract and early H₂S prodrugs, reported modest reductions in blood pressure and arterial stiffness, although clinical evidence remains limited and heterogeneous. Existing literature supports a biologically plausible role for H₂S in hypertension and suggests that H₂S-enhancing interventions may offer therapeutic benefit. However, translation to clinical practice is hindered by small sample sizes, heterogeneity of study designs, and lack of standardized H₂S measurement. Future research should prioritize validated biomarkers and adequately powered clinical trials.

## Introduction and background

Hypertension affects an estimated 1.28 billion people worldwide and remains a major cause of morbidity and mortality from cardiovascular, renal, and cerebrovascular disease [1]. Despite the availability of evidence-based pharmacotherapy, a large and disproportionate number of patients remain uncontrolled and resistant to treatment. This has increased interest in searches for new, mechanism-based adjunct therapies capable of reducing endothelial dysfunction, oxidative stress, and vascular inflammation, thus improving blood pressure control [2].

Endogenously produced gasotransmitters help regulate vascular homeostasis and blood pressure. Besides nitric oxide and carbon monoxide, hydrogen sulfide (H₂S) has also come into focus as another gasotransmitter notable for its vasodilatory, antioxidant, and anti-inflammatory properties in blood pressure regulation [3]. H₂S acts largely through promoting membrane hyperpolarization and associated vasorelaxation involving adenosine triphosphate (ATP)-sensitive potassium-channel activation and endothelium-derived hyperpolarizing-factor-like action [4]. It also acts bidirectionally with the nitric oxide signaling pathway through mechanisms involving endothelial nitric oxide synthase (eNOS) S-sulfhydration and downstream signaling processes [5]. H₂S also regulates mitochondrial function and the cellular redox state [6].

Preclinical studies provide strong support for the role of H₂S in blood pressure regulation. Genetic deletion of cystathionine γ-lyase (CSE), a key H₂S-producing enzyme, leads to spontaneous hypertension and endothelial dysfunction, while restoration of H₂S through gene transfer or donor compounds lowers blood pressure, improves vascular reactivity, and attenuates oxidative stress and adverse remodeling [7]. Human data, though more limited, echo these findings: lower circulating H₂S and altered sulfide metabolites have been associated with higher blood pressure, greater arterial stiffness, and reduced vasodilatory capacity, with effects that are accentuated by aging [8-10]. What remains less certain, however, is how these observations can be translated into routine clinical care. The purpose of this review is to synthesize preclinical and human evidence linking H₂S to the pathophysiology of hypertension, to evaluate the current state of pharmacological and nutritional H₂S-based interventions, and to identify the key challenges that must be addressed before translation into clinical practice [11].

## Review

Materials and methods

This review was conducted following the Preferred Reporting Items for Systematic Reviews and Meta-Analyses extension for Scoping Reviews (PRISMA-ScR) checklist, which is appropriate for mapping broad, heterogeneous evidence across mechanistic, preclinical, and clinical domains. A scoping review approach was chosen to capture the diversity of study designs in the hydrogen sulfide (H₂S) literature, including mechanistic studies, animal experiments, human investigations, and secondary syntheses, and to identify knowledge gaps relevant to hypertension research. The review protocol was not prospectively registered.

Information Sources and Search Strategy

Comprehensive searches were conducted in PubMed for English-language publications from January 1, 2001, to July 31, 2025, with a final update performed on August 10, 2025. Supplementary sources included Google Scholar (first 300 relevance-ranked results screened), ClinicalTrials.gov, and targeted hand-searching of reference lists. Search strategies combined controlled vocabulary and free-text terms related to H₂S biology (e.g., “hydrogen sulfide,” H₂S, cystathionine γ-lyase/CSE, cystathionine β-synthase (CBS), 3-mercaptopyruvate sulfurtransferase (3-MST), sodium hydrosulfide (NaHS), GYY4137, diallyl trisulfide, aged garlic extract) with hypertension- and vascular-related terms (e.g., hypertension, high blood pressure, arterial stiffness, endothelial function, pulse wave velocity, flow-mediated dilation). Exact Boolean queries for each source are provided in Appendix A.

Eligibility Criteria

Eligible sources included: Mechanistic studies examine molecular, enzymatic, or signaling pathways linking H₂S to vascular or renal regulation relevant to blood pressure. Preclinical animal studies investigate endogenous H₂S, genetic deletion of H₂S-generating enzymes, or exogenous H₂S donors in models of hypertension or vascular dysfunction. Observational human studies assess H₂S biomarkers, metabolic pathways, endothelial function, or hemodynamic parameters. Interventional human studies include randomized controlled trials, non-randomized trials, and early-phase studies targeting H₂S pathways or evaluating H₂S-enhancing interventions (e.g., aged garlic extract, SG1002). Narrative reviews, systematic reviews, meta-analyses, and guidelines relevant to H₂S and blood pressure were included for contextual mapping only, consistent with the principles of scoping review methodology.

Exclusion Criteria

They were non-English publications; unavailable full text; purely in vitro studies without vascular or hypertensive relevance; unrelated to blood pressure, vascular physiology, or H₂S pathways; or overlapping datasets. Records indexed in PubMed with English-language bibliographic information and sufficient English-language abstract-level data were retained when the relevant outcomes could be extracted reliably.

Study Selection

All records were imported and de-duplicated. Screening proceeded in two stages: (1) title/abstract screening to determine potential relevance and (2) full-text review to confirm eligibility. Reasons for exclusion at the full-text stage were documented. Both reviewers independently assessed eligibility, and disagreements were resolved through discussion.

Data Charting

Data were charted using a standardized extraction framework that included: study design; model or population characteristics; exposure, intervention, or pathway evaluated; mechanistic endpoints; and vascular or blood pressure-related outcomes. Registry records were consulted to confirm design and population details for clinical trials.

Results

A total of 873 records were identified across all sources (PubMed = 570; Google Scholar = 300 relevance-ranked records; ClinicalTrials.gov = 3). After removal of 223 duplicates, 650 unique records were screened at the title/abstract level. Of these, 581 records were excluded for not meeting eligibility criteria. A total of 69 full texts were reviewed. Thirty records were excluded for the following reasons: incorrect population or setting (n = 9); absence of blood pressure or vascular endpoints (n = 8); mechanistic studies unrelated to hypertension (n = 6); inadequate or unclear data (n = 4); or duplicate/overlapping reports (n = 3).

**Figure 1:**
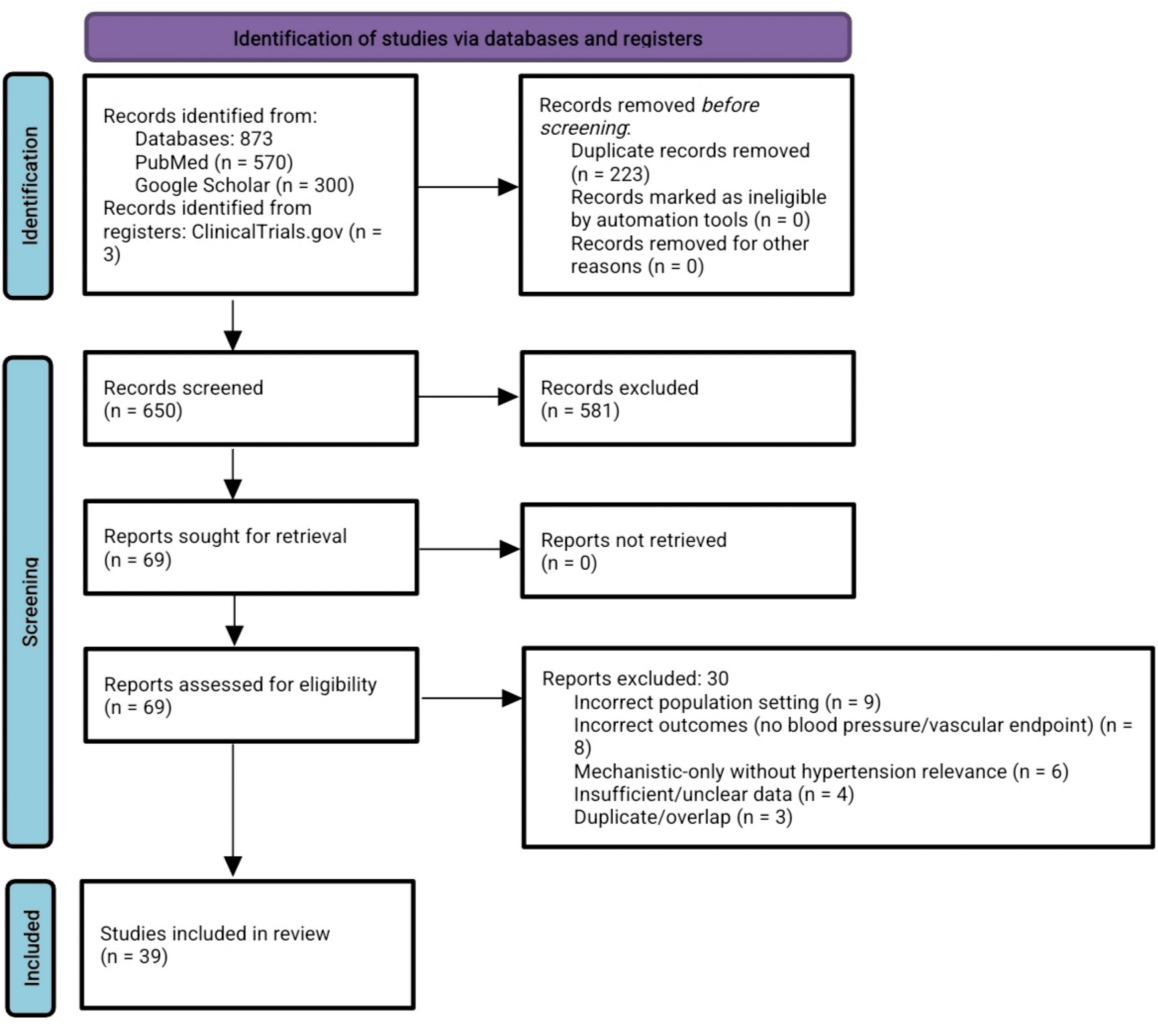
PRISMA-ScR flow diagram of study selection PRISMA-ScR: Preferred Reporting Items for Systematic Reviews and Meta-Analyses extension for Scoping Reviews.

In total, 39 sources were included, categorized into three groups consistent with scoping review methodology: Primary experimental evidence (n = 12): mechanistic and preclinical animal studies evaluating H₂S pathways or donor administration in hypertensive or vascular models. Primary human evidence (n = 9): observational studies, randomized controlled trials, and non-randomized interventional studies examining circulating H₂S, vascular function, or H₂S-enhancing therapies. Contextual secondary literature (n = 18): narrative reviews, systematic reviews, meta-analyses, and guidelines providing background or interpretive context. These were not treated as primary data sources and were extracted separately. The study selection process is illustrated in Figure 1. The characteristics of the primary experimental sources are summarized in Table 1 [4,6,7,12-20], primary human sources are summarized in Table 2 [8-10,21-26], and contextual secondary sources are listed in Table 3 [1-3,5,11,27-39].

**Table 1:** Mechanistic and preclinical studies evaluating hydrogen sulfide pathways in hypertension 3-MST: 3-mercaptopyruvate sulfurtransferase; Ang II: angiotensin II; BMP4: bone morphogenetic protein 4; BP: blood pressure; CBS: cystathionine β-synthase; COX-2: cyclooxygenase-2; CSE: cystathionine γ-lyase; eNOS: endothelial nitric oxide synthase; H₂S: hydrogen sulfide; KATP: ATP-sensitive potassium channel; NaHS: sodium hydrosulfide; NO: nitric oxide; SHR: spontaneously hypertensive rat; TGF-β: transforming growth factor beta.

Author (Year)	Model / System	Exposure / Intervention	Mechanistic Endpoints	Key Findings
Zhao et al. (2001) [4]	Rat thoracic aorta	Exogenous H₂S	KATP channel signaling	H₂S induced dose-dependent vasorelaxation via KATP channel activation.
Yan et al. (2004) [15]	Spontaneously hypertensive rats	NaHS administration	BP regulation, vascular relaxation	Lower H₂S was implicated in spontaneous hypertension; NaHS lowered arterial pressure and improved relaxation.
Li et al. (2008) [18]	Rat aorta and cultured vascular cells	GYY4137 (slow-release donor)	Release kinetics, vasorelaxation	GYY4137 produced sustained H₂S release with prolonged vasorelaxant and cytoprotective effects.
Yang et al. (2008) [7]	CSE knockout mice	Genetic deletion of CSE	Endothelium-dependent relaxation	CSE deficiency caused spontaneous hypertension and impaired endothelium-dependent vasodilation.
Coletta et al. (2012) [19]	Cultured endothelial cells and isolated arteries	Modulation of H₂S and NO pathways	H₂S-NO crosstalk	H₂S and NO were mutually dependent in endothelium-dependent vasorelaxation and angiogenesis.
Sen et al. (2012) [12]	Rat renal arteries (hyperhomocysteinemia model)	CBS/CSE/3-MST gene therapy	Renovascular relaxation	Enhanced endogenous H₂S generation improved ex vivo renovascular relaxation.
Altaany et al. (2013) [20]	Human endothelial cells	H₂S-NO interaction experiments	eNOS activity, S-sulfhydration	H₂S enhanced eNOS activity and NO production via S-sulfhydration mechanisms.
Kondo et al. (2013) [6]	Mouse pressure-overload model	NaHS administration	eNOS/NO bioavailability	H₂S attenuated hypertrophy and dysfunction with upregulation of eNOS and increased NO bioavailability.
Al-Magableh et al. (2015) [16]	Angiotensin II-induced hypertensive mice	Chronic NaHS treatment	Oxidative stress markers	H₂S reduced BP and oxidative stress in Ang II-induced hypertension.
Sun et al. (2014) [14]	Spontaneously hypertensive rats	NaHS supplementation	TGF-β/Smad signaling, fibrosis	H₂S reduced myocardial collagen remodeling by inhibiting TGF-β/Smad signaling.
Xiao et al. (2016) [13]	Spontaneously hypertensive rats	H₂S donor treatment	BMP4/COX-2, oxidative stress	H₂S improved endothelial function and reduced BP via BMP4/COX-2 pathway suppression.
Macabrey et al. (2022) [17]	Endothelial cells + in vivo neovascularization model	Sodium thiosulfate	Endothelial proliferation, angiogenesis	Sodium thiosulfate promoted endothelial proliferation and neovascularization as a clinically practical H₂S source.

**Table 2:** Human observational and interventional studies evaluating H₂S biology and therapies in hypertension ACE: angiotensin-converting enzyme; BP: blood pressure; H₂S: hydrogen sulfide; NO: nitric oxide; AGE, aged garlic extract; GarGIC, Garlic in Gastrointestinal Cardiovascular trial; PWV, pulse wave velocity; PK, pharmacokinetics; PD, pharmacodynamics.

Author (Year)	Study Type	Population	Exposure / Intervention	Outcomes	Key Findings
Chen et al. (2007) [23]	Observational (cross-sectional)	Children with essential hypertension	Circulating H₂S, homocysteine	BP	Reduced plasma H₂S and increased homocysteine were observed in pediatric hypertension.
Sun et al. (2007) [21]	Observational (cross-sectional)	Hypertensive adults	Circulating H₂S, homocysteine	BP; complications	Lower H₂S and higher homocysteine were associated with higher BP and complications.
Ried et al. (2010) [9]	Randomized controlled trial	Treated but uncontrolled hypertensives	Aged garlic extract	BP	AGE significantly lowered BP compared with placebo over 12 weeks.
Ried et al. (2013) [24]	Randomized controlled trial	Hypertensive adults	Aged garlic extract (dose-response)	BP	Dose-dependent BP reduction with optimal response around 480 mg/day.
Polhemus et al. (2015) [10]	Phase 1/2 clinical trial	Heart failure patients + healthy volunteers	SG1002 (oral H₂S prodrug)	Safety; plasma sulfide; NO biomarkers; BP	SG1002 was well tolerated and increased plasma sulfide and nitrite/nitrate; BP unchanged (short duration).
Greaney et al. (2017) [22]	Physiological/observational	Adults (HTN vs normotensive controls)	H₂S-mediated vasodilation assessment	Microvascular function	Hypertensive adults showed impaired H₂S-mediated vasodilation contributing to endothelial dysfunction.
Ried et al. (2018) [25]	Randomized controlled trial	Hypertensive adults (GarGIC trial)	Aged garlic extract	BP; PWV; inflammatory markers; microbiota	AGE reduced BP and arterial stiffness and improved microbiota/inflammatory markers.
ClinicalTrials.gov record (2020) [26]	Registry entry	Heart failure patients	SG1002 (NCT01989208)	Safety/PK/PD	Registry details confirm ongoing evaluation of safety and pharmacodynamic endpoints.
Dillon et al. (2021) [8]	Observational	Adults with hypertension (treated)	Sulfhydryl-donating antihypertensive therapy	Microvascular vasodilation	Chronic thiol-donor therapy improved H₂S-dependent microvascular vasodilation.

**Table 3:** Secondary literature (narrative reviews, systematic reviews/meta-analyses, guidelines) H₂S: hydrogen sulfide; CV, cardiovascular; HTN, hypertension; AGE, aged garlic extract; PWV, pulse wave velocity; CVD, cardiovascular disease.

Author (Year)	Type	Scope	Key Contribution
Ried et al. (2008) [27]	Systematic review/meta-analysis	Garlic and BP	Meta-analysis showing clinically relevant BP reductions in hypertensive subjects.
Whiteman et al. (2011) [11]	Narrative review	Biomarkers/tools	Critical appraisal of biomarkers and pharmacological tools for H₂S research.
Olson (2012) [3]	Narrative review	H₂S chemistry/biology	Highlights chemical speciation and measurement challenges.
Stabler et al. (2012) [28]	Cochrane systematic review	Garlic and CV outcomes	Assesses evidence quality; notes modest BP effect and limited outcome data.
Mani et al. (2014) [30]	Narrative review	Atherosclerosis	Links H₂S signaling with vascular protection and anti-atherogenic mechanisms.
Zhao et al. (2014) [38]	Narrative review	H₂S-releasing agents	Chemistry and biological applications of H₂S donors.
Bełtowski (2015) [35]	Narrative review	Pharmacology	Update on donor classes, targets, and safety considerations.
Meng et al. (2015) [5]	Narrative review	H₂S in hypertension	Synthesizes mechanistic and translational evidence in HTN.
Olson (2015) [34]	Narrative review	O₂ sensing	Describes H₂S as an oxygen-dependent modulator of vascular response.
Cacanyiova et al. (2016) [32]	Narrative review	Blood pressure regulation	Summarizes H₂S roles in vascular tone and BP regulation.
Li et al. (2018) [36]	Narrative review	Therapeutic development	Tracks evolution of H₂S therapeutics toward clinical application.
Ried (2020) [29]	Review/meta-analysis	AGE, arterial stiffness, microbiota	Updated synthesis including PWV and microbiota-related outcomes.
Gojon and Morales (2020) [33]	Narrative review	H₂S prodrugs	Review of SG1002 and related prodrug chemistry/pharmacology.
Wang et al. (2021) [37]	Narrative review	Donors in CVD	Summarizes evidence for H₂S donors in cardiovascular diseases.
Kolluru et al. (2023) [31]	Narrative review	Cardiovascular function	Comprehensive overview of sulfide signaling in cardiovascular disease.
Wang (2010) [2]	Narrative review	H₂S as gasotransmitter	Overview of H₂S biology and medical relevance.
Wang (2023) [39]	Narrative review	H₂S in hypertension	Updated synthesis of H₂S roles in HTN development and complications.
WHO (2023) [1]	Guideline / factsheet	Global hypertension burden	Provides epidemiology and key public health messages on hypertension.

Preclinical and Mechanistic Studies

A total of 12 experimental studies investigated the cardiovascular effects of H₂S pathways in preclinical models of hypertension. These included both mechanistic studies, which present specific signaling pathways and molecular mediators, and functional studies, which demonstrated physiological outcomes such as reduction of blood pressure, improvement of endothelial function, or diminishment of oxidative stress (Table 1). These studies show a consistent relationship between reduced H₂S bioavailability and the development of hypertension, endothelial dysfunction, and adverse vascular remodeling.

The studies showed a clear link between low H₂S bioavailability and the development of hypertension, endothelial dysfunction, and vascular remodeling. H₂S promoted vasorelaxation through activation of KATP channels and endothelium-derived hyperpolarizing-factor-like mechanisms [4]. Nitric oxide bioavailability was improved by upregulating endothelial nitric oxide synthase during pressure overload [6]. CSE knockout led to spontaneous hypertension and dysfunctional vasodilation, confirming its essential role in the synthesis of H₂S [7]. Gene therapy targeting H₂S-producing enzymes (CBS, CSE, 3-MST) enhanced renovascular relaxation in hyperhomocysteinemia [10]. Other studies on signaling showed that H₂S downregulated the bone morphogenetic protein 4 (BMP4)/cyclooxygenase-2 (COX-2) and transforming growth factor beta (TGF)-β/Smad pathways, protecting against endothelial and myocardial injury [13,14]. Functional experiments proved that decreased H₂S was responsible for elevated blood pressure in spontaneously hypertensive rats [15].

Exogenous H₂S supplementation also yielded positive effects. Fast-releasing donors such as NaHS reduced mean arterial pressure and oxidative stress [16]. Endothelial proliferation and neovascularization were stimulated by sodium thiosulfate [17]. Slow-releasing donors, like GYY4137, provided sustained antioxidant and anti-inflammatory activity [18]. Crosstalk between H₂S and NO signaling further enhanced vasorelaxation and angiogenesis [19,20]. Available data support the idea that H₂S acts as a regulator of vascular tone and could be a potential therapeutic target in hypertension.

Human Observational Evidence

Four observational studies assessed circulating H₂S and vascular function in human populations (Table 2). In adults with hypertension, impaired H₂S-mediated vasodilation was identified as a cause of microvascular endothelial dysfunction [21]. Plasma H₂S levels were consistently lower in patients with hypertension compared with normotensive controls, both in adults and in children [22,23]. These reductions were often accompanied by elevated homocysteine levels, suggesting a disruption of the endogenous sulfur-amino-acid metabolic pathway.

Decreased H₂S bioavailability also correlated with increased arterial stiffness and diminished responsiveness to exogenous H₂S donors [21]. Importantly, chronic treatment with sulfhydryl-donating antihypertensive drugs partially reversed microvascular vasodilation, supporting the physiological role of H₂S signaling in human blood pressure regulation [8]. Human studies show that impaired endogenous H₂S synthesis and signaling are linked to vascular stiffness, endothelial dysfunction, and the pathogenesis of hypertension.

Interventional Studies

Four completed human trials and one registered study assessed approaches to enhance H₂S bioavailability (Table 3). Three randomized controlled trials tested aged garlic extract (AGE), a nutritional source of organosulfur compounds, in adults with treated but uncontrolled hypertension [22,24,25]. Each of these was a double-blind and placebo-controlled study. AGE supplementation decreased systolic and diastolic blood pressure by about 5-10 mmHg and reduced arterial stiffness. Secondary benefits included better endothelial function, decreased inflammatory markers, and improved gut microbiological diversity.

A Phase 1 clinical trial investigated the novel oral H₂S prodrug SG1002 in heart failure patients and in healthy volunteers [23]. Over one week of dosing (200-800 mg twice daily), SG1002 induced a dose-dependent increase of plasma sulfide, nitrite/nitrate, and cyclic guanosine monophosphate (cGMP), confirming enhanced H₂S-NO signaling. Treatment was well tolerated, with no serious adverse events or hypotension. Although blood pressure remained unchanged due to short duration, the study established safety, oral bioavailability, and mechanistic activation of H₂S pathways in humans. An ongoing registry study is further characterizing its safety and pharmacodynamic profile [26]. These studies demonstrate that both nutritional and pharmacologic interventions can safely improve H₂S signaling in humans to enhance vascular compliance and support its potential role in treating hypertension.

Risk-of-Bias Assessment

**Figure 2:**
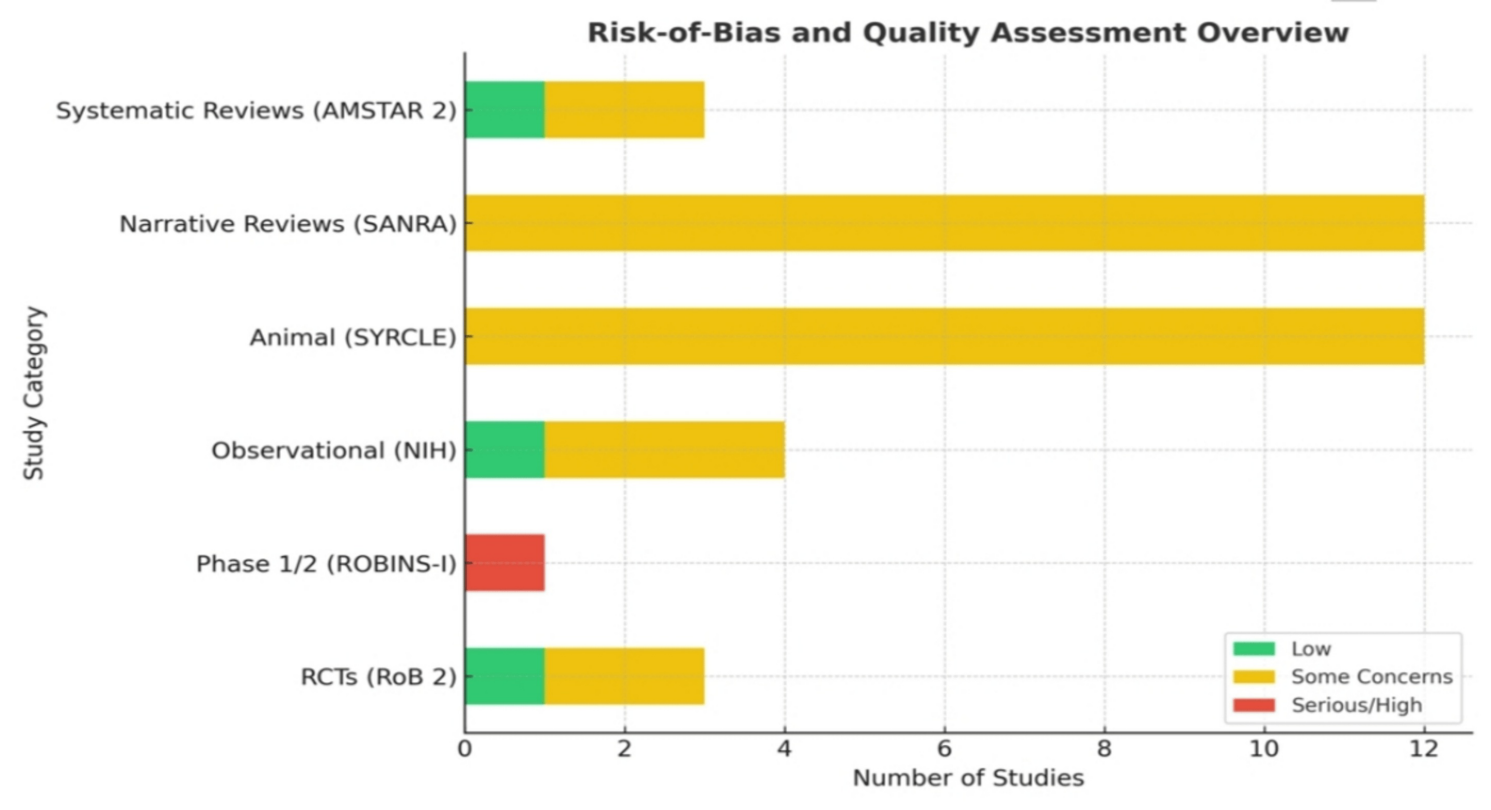
Traffic-light summary of risk-of-bias and quality assessment across included studies AMSTAR 2, A MeaSurement Tool to Assess Systematic Reviews 2; SANRA, Scale for the Assessment of Narrative Review Articles; SYRCLE, Systematic Review Centre for Laboratory Animal Experimentation; ROBINS-I, Risk Of Bias In Non-randomized Studies of Interventions; RoB 2, Revised Cochrane risk-of-bias tool for randomized trials; RCT, randomized controlled trial.

Because scoping reviews aim to map available evidence rather than evaluate effectiveness, risk-of-bias assessments were summarized descriptively. Appropriate tools were applied for each study type: Systematic Review Centre for Laboratory Animal Experimentation (SYRCLE) [40] for animal studies, NIH Quality Assessment Tool [41] for observational studies, Revised Cochrane risk-of-bias tool for randomized trials (RoB 2) [42] for randomized controlled trials, Risk Of Bias In Non-randomized Studies of Interventions (ROBINS-I) [43] for non-randomized trials, Scale for the Assessment of Narrative Review Articles (SANRA) [44] for narrative reviews, and A MeaSurement Tool to Assess Systematic Reviews 2 (AMSTAR 2) [45] for systematic reviews. Results are presented in tabular format by study category, and findings are summarized in Tables 4-9. The randomized trials of aged garlic extract were rated as low risk or some concerns, mainly because of incomplete reporting of randomization or protocol transparency in earlier studies. The early-phase SG1002 study was rated as a serious overall risk of bias because it was open-label and lacked a comparator arm. Observational studies were generally rated as fair to good, while animal and narrative review evidence was retained mainly for contextual mapping because reporting of randomization, blinding, or review methodology was often incomplete. Figure 2 provides a visual summary of the distribution of overall judgments across study categories.

**Table 4:** Cochrane RoB 2: randomized controlled trials Low: low risk of bias; SC: some concerns; RoB 2, Revised Cochrane risk-of-bias tool for randomized trials.

Study (Year)	Randomization Process	Deviations From Intended Interventions	Missing Outcome Data	Measurement of Outcome	Selection of Reported Result	Overall	Notes / Justification
Ried et al., 2010 [9]	SC	Low	Low	Low	SC	SC	Randomized, blinded, but allocation concealment and preregistration not clearly reported.
Ried et al., 2013 [24]	SC	Low	Low	Low	SC	SC	Adequate blinding and follow-up; limited protocol transparency and unclear randomization method.
Ried et al., 2018 [25]	Low	Low	Low	Low	Low	Low	Fully randomized, double-blind, preregistered with complete outcome reporting.

**Table 5:** ROBINS-I: non-randomized/early-phase interventional studies Low: low risk of bias; SC: some concerns; ROBINS-I, Risk Of Bias In Non-randomized Studies of Interventions.

Study (Year)	Confounding	Selection of Participants	Classification of Interventions	Deviations From Intended Interventions	Missing Data	Measurement of Outcomes	Selection of Reported Results	Overall	Notes / Justification
Polhemus et al., 2015 [10]	Serious	SC	Low	Low	Low	Low	SC	Serious	Open-label, no comparator arm; objective biochemical outcomes mitigate but do not remove confounding.

**Table 6:** NIH quality assessment: observational cohort/cross-sectional studies NA: not applicable; NR: not reported. Partial indicates confounders measured but not comprehensively adjusted.

Study (Year)	Clear Research Question	Defined Population	Participation Rate ≥50%	Sample Size Justification	Exposure Measured Prior to Outcome (If Applicable)	Confounders Measured/Adjusted	Outcome Measures Valid/Reliable	Blinding of Outcome Assessors	Missing Data Handled	Overall	Notes / Justification
Chen et al., 2007 [23]	Yes	Yes	NR	No	NA	Partial	Yes	Yes	NR	Fair	Pediatric cohort; partial control of confounders; small N.
Sun et al., 2007 [21]	Yes	Yes	NR	No	NA	Partial	Yes	NR	NR	Fair	Cross-sectional; limited multivariable analysis; acceptable outcome validity.
Greaney et al., 2017 [22]	Yes	Yes	NR	Yes	NA	Partial	Yes	NR	NR	Fair	Mechanistic design; high measurement validity; small N.
Dillon et al., 2021 [8]	Yes	Yes	NR	No	NA	Partial	Yes	NR	Yes	Good	Adequate design; small sample; limited adjustment for confounders.

**Table 7:** SYRCLE risk of bias: animal/experimental studies All included animal studies (n = 12, 2001-2022) exhibited similar design and reporting features and were therefore appraised collectively. Individual ratings did not differ substantially across studies. NI: not informed; NR: not reported; SC: some concerns; SYRCLE, Systematic Review Centre for Laboratory Animal Experimentation.

Study (Year)	Sequence Generation	Baseline Similarity	Allocation Concealment	Random Housing	Blinding Caregivers	Random Outcome Assessment	Blinding Outcome Assessors	Incomplete Outcome Data	Selective Reporting	Other Bias	Overall	Notes / Justification
Zhao et al., 2001 [4] to Macabrey et al., 2022 [17] (12 studies, grouped)	NI/NR	Low	NI/NR	SC	SC	Low	SC	Low	Low	None	SC	Consistent experimental controls and full reporting; randomization/blinding generally unreported.

**Table 8:** SANRA: narrative reviews (contextual, not used for effect estimates) Narrative reviews (n = 14) were evaluated as a group given their comparable structure and reporting style; all received comparable SANRA domain scores. SANRA, Scale for the Assessment of Narrative Review Articles.

Review (Year)	Aim Justified (0-2)	Literature Search Stated (0-2)	Referencing (0-2)	Scientific Reasoning (0-2)	Appropriate Data Presentation (0-2)	Overall	Notes / Justification
Wang, 2010 [2] to Wang, 2023 [39] (14 studies, grouped)	2	2	1	2	2	9	Logical structure, relevant citations; minor referencing limits; conclusions aligned with data.

**Table 9:** AMSTAR 2: systematic reviews and meta-analyses (contextual) SR, systematic review; MA, meta-analysis; AMSTAR 2, A MeaSurement Tool to Assess Systematic Reviews 2.

SR/MA (Year)	Protocol Registered	Adequate Search	Dual Screening/Extraction	Risk of Bias Considered	Methods Appropriate	Publication Bias Assessed	Overall Confidence	Notes / Justification
Ried et al., 2008 [27]	No	Yes	Yes	Partial	Yes	Partial	Moderate	Limited bias appraisal and incomplete search reporting in early trials.
Stabler et al., 2012 [28]	Yes	Yes	Yes	Yes	Yes	Yes	Moderate	Standardized Cochrane process with transparent bias assessment and reproducibility.
Ried, 2020 [29]	Partial	Yes	Yes	Partial	Yes	Partial	Moderate	Up-to-date meta-analysis with predefined criteria and comprehensive inclusion.

Justification for Exclusion of Registry and Guideline/Factsheet From Risk-of-Bias Assessment

The ClinicalTrials.gov registry record (NCT01989208) and the WHO 'Hypertension: Key Facts' fact sheet were excluded from formal risk-of-bias assessment because they do not constitute primary or secondary research studies with analyzable data. Registry entries provide administrative information on study design and status but lack methodological details needed for bias appraisal (e.g., randomization, blinding, data handling). Similarly, the WHO fact sheet is a global epidemiological summary and policy document that lacks a defined study design or specific outcomes. Applying tools such as RoB 2, ROBINS-I, or SANRA would therefore be methodologically inappropriate. It was included only as contextual background to describe the global burden of hypertension.

Excluding these items from bias scoring ensures methodological consistency and transparency while maintaining focus on empirical evidence directly relevant to hydrogen sulfide and hypertension. Instead, they were narratively assessed using predefined criteria: (i) authorship/source authority, (ii) date of publication or last update, (iii) completeness and clarity of information provided (e.g., endpoints, sample size, or epidemiologic scope), and (iv) consistency with the corresponding peer-reviewed evidence. These items were retained for context but were not weighted in synthesis.

Both sources met the predefined criteria for authority, recency, and internal consistency. The WHO fact sheet was confirmed to be the most recent global epidemiologic summary (updated 2023), and the registry record verified trial registration and design details for SG1002. Neither provided analyzable outcomes and were therefore used solely as contextual references.

Discussion

Since research on H₂S ranges from mechanistic experiments to early human trials, and the field is still developing, a scoping review allowed us to describe the full landscape of existing evidence without forcing a quantitative synthesis. The distribution of study designs and key characteristics is summarized in Tables 1-3. This scoping review synthesized preclinical, observational, and interventional evidence to clarify how hydrogen sulfide (H₂S) contributes to the development and modulation of hypertension. Across experimental models, reduction of endogenous H₂S consistently resulted in higher blood pressure, increased vascular tone, and impaired endothelial reactivity. These effects were observed in spontaneously hypertensive rats, in CSE-deficient mice, and in angiotensin II-driven hypertension models, with restoration of H₂S signaling improving endothelial function and lowering blood pressure. Although methodological details were often incomplete, the direction of effect was uniform across all 12 preclinical studies, supporting a mechanistic role for H₂S in blood pressure regulation through interactions with nitric oxide pathways, ATP-sensitive potassium channel (KATP) channel activation, reduction of oxidative stress, and suppression of pro-inflammatory vascular remodeling [7,13-17,21].

The human observational data aligned with these mechanistic findings. Adults with hypertension consistently demonstrated that impaired H₂S signaling was associated with microvascular dysfunction [19] and lower circulating or whole-blood H₂S levels than normotensive controls [20]. These associations support the concept that H₂S deficiency contributes to early vascular abnormalities rather than being only a secondary finding of established hypertension.

Interventional evidence in humans was more limited but provided important insights. Randomized trials of aged garlic extract (AGE) demonstrated modest reductions in systolic blood pressure and, in some cases, improvements in arterial stiffness [22,24,25], consistent with enhanced endogenous H₂S bioavailability through organosulfur compounds. By contrast, the early-phase SG1002 trial increased circulating sulfide biomarkers without reducing blood pressure during short follow-up [23]. These discrepancies likely reflect differences in formulation, duration of exposure, dose, and trial design. Overall, while the interventional data support the biological plausibility suggested by preclinical studies, they remain insufficient to draw firm clinical conclusions.

Taken together, the evidence shows a coherent pattern in which impaired H₂S production or signaling contributes to the hypertensive phenotype, while restoration of sulfide bioavailability appears beneficial. However, translation of these findings into clinical practice remains incomplete. A major barrier is the absence of standardized, clinically validated assays for H₂S measurement, which hampers comparability across studies and limits biomarker-based risk stratification. Existing human trials are small, heterogeneous, and short in duration, leaving unanswered questions about optimal dosing strategies, safety, and which patient populations might benefit most from H₂S-modulating therapies.

Future research should prioritize the development of standardized analytical methods for H₂S quantification and conduct larger, well-designed clinical trials that include mechanistic vascular endpoints. Improved vascular and metabolic phenotyping may help identify individuals in whom H₂S deficiency is a driver of hypertension. While current evidence supports the biological relevance of H₂S in vascular regulation, substantial methodological and translational gaps must be addressed before H₂S-targeted interventions can be integrated into routine hypertension management.

Limitations

This review also possesses several limitations that merit consideration at the time of interpreting existing evidence. Most human studies that have evaluated H₂S and blood pressure regulation had small patient populations, short study durations, and no uniformity in study design, often with a surrogacy for vascular or biochemical endpoints instead of hard cardiovascular events. Preclinical studies had a variety of animal models, donor entities, and dosing schemes, making them less comparable and less transferable to humans.

Quantitation of H₂S is technically problematic: current methods differ significantly in their extent of sensitivity, specificity, and capability to discriminate free, bound, and acid-labile sulfide pools, leading to variability in biomarker results across studies. Also, interindividual variations in enzyme activities, redox status, and sulfur-amino acid metabolism can affect responses to H₂S-modulator therapy, and such factors are not typically addressed.

The review was limited to English-language publications and studies that have been recorded in PubMed, Google Scholar, ClinicalTrials.gov, and hand-searching. Because Embase, Cochrane CENTRAL, Scopus, and Web of Science were not searched, relevant studies indexed exclusively in these databases may have been missed. Overall, these factors highlight the necessity for standardized protocols for measuring quantities, consistent experimental designs, and adequately powered clinical studies before a definitive conclusion regarding the therapeutic efficacy of H₂S can be established in the clinical setting of hypertension.

## Conclusions

This review highlights the role of H₂S in maintaining vascular health, endothelial function, and regulating blood pressure. Its ability to promote vasodilation, reduce oxidative stress, and limit vascular remodeling makes it a promising target for new approaches to hypertension treatment. Early studies suggest that restoring H₂S balance may help lower blood pressure and improve endothelial function, offering potential benefits for patients with uncontrolled hypertension despite therapy or marked endothelial dysfunction.

However, the available clinical evidence is still limited. Most studies have involved small groups of participants and short follow-up periods, making it difficult to draw firm conclusions about long-term safety and effectiveness. Future research should focus on developing reliable methods to measure H₂S activity, improving donor compounds for more consistent delivery, and conducting large, well-controlled trials to confirm its clinical value. Should we manage to address these gaps, H₂S-based therapies can be evaluated for integration into the present evidence-based hypertension treatment.

## Appendices

Appendix A

Search Strings

Scope and dates: Searches covered January 1, 2001, to July 31, 2025 (English only). Final search update: August 10, 2025.

PubMed

Exact Boolean string used (copy/paste):

("hydrogen sulfide"[Title/Abstract] OR H2S[Title/Abstract]

OR "cystathionine gamma-lyase"[Title/Abstract]

OR "cystathionine beta-synthase"[Title/Abstract]

OR "3-mercaptopyruvate sulfurtransferase"[Title/Abstract]

OR NaHS[Title/Abstract] OR GYY4137[Title/Abstract]

OR "diallyl trisulfide"[Title/Abstract]

OR "aged garlic extract"[Title/Abstract])

AND

(hypertension[Title/Abstract] OR "high blood pressure"[Title/Abstract]

OR "arterial stiffness"[Title/Abstract]

OR "pulse wave velocity"[Title/Abstract]

OR "endothelial function"[Title/Abstract]

OR "flow-mediated dilation"[Title/Abstract])

AND

("2001/01/01"[Date - Publication] : "2025/07/31"[Date - Publication])

AND

(English[Language])

Limits applied: English; Publication date January 1, 2001, to July 31, 2025. Field tags: Title/Abstract for all terms. Result count retrieved: 570 records.

Notes for replication: On PubMed, apply the date range in Filters → Publication dates and Language → English; ensure Best Match/Relevance does not alter inclusion (all 570 were screened).

Google Scholar

Exact Boolean string used (copy/paste):

("hydrogen sulfide" OR H2S OR "cystathionine gamma-lyase"

OR "cystathionine beta-synthase"

OR "3-mercaptopyruvate sulfurtransferase"

OR NaHS OR GYY4137 OR "diallyl trisulfide"

OR "aged garlic extract")

AND

(hypertension OR "high blood pressure"

OR "arterial stiffness" OR "pulse wave velocity"

OR "endothelial function" OR "flow-mediated dilation")

Filters applied in Google Scholar (GS): Custom date range 2001-2025; language English (where available). Raw hits returned: ~19,700. Prespecified screening rule: Screened only the first 300 relevance-ranked results (titles/abstract snippets), consistent with feasibility and Preferred Reporting Items for Systematic Reviews and Meta-Analyses literature search extension (PRISMA-S) guidance for Google Scholar.

Trial registries

ClinicalTrials.gov search string:

("hydrogen sulfide" OR H2S OR NaHS OR GYY4137 OR "diallyl trisulfide" OR "aged garlic extract")

AND

(hypertension OR "high blood pressure" OR "vascular stiffness")

Hits returned: 3 relevant trials, including NCT01989208 (SG1002 Phase 1 safety/pharmacology study). Purpose: Identify registered or ongoing human studies; confirm whether included clinical evidence had prior registration.

Other sources (hand-search)

• WHO factsheet: Hypertension: key facts (accessed July 2025) via who.int [1].

De-duplication and management (for transparency)

• Records taken forward to de-duplication: PubMed (570) + Google Scholar (first 300) + ClinicalTrials.gov (3) = 873 total records.

• Duplicates removed: 223 (manual match by title ± first author/year).

• Unique records screened (title/abstract): 650.

• Full-text assessed: 69.

• Included in qualitative synthesis: 39 studies.
